# Supplementary figures and images for: Transcriptome analysis of the eggs of the silkworm pale red egg (rep-1) mutant at 36 hours after oviposition
Source: PLoS One. 2020 Aug 7;15(8):e0237242. doi: 10.1371/journal.pone.0237242 (PMC7413551; doi:10.1371/journal.pone.0237242)

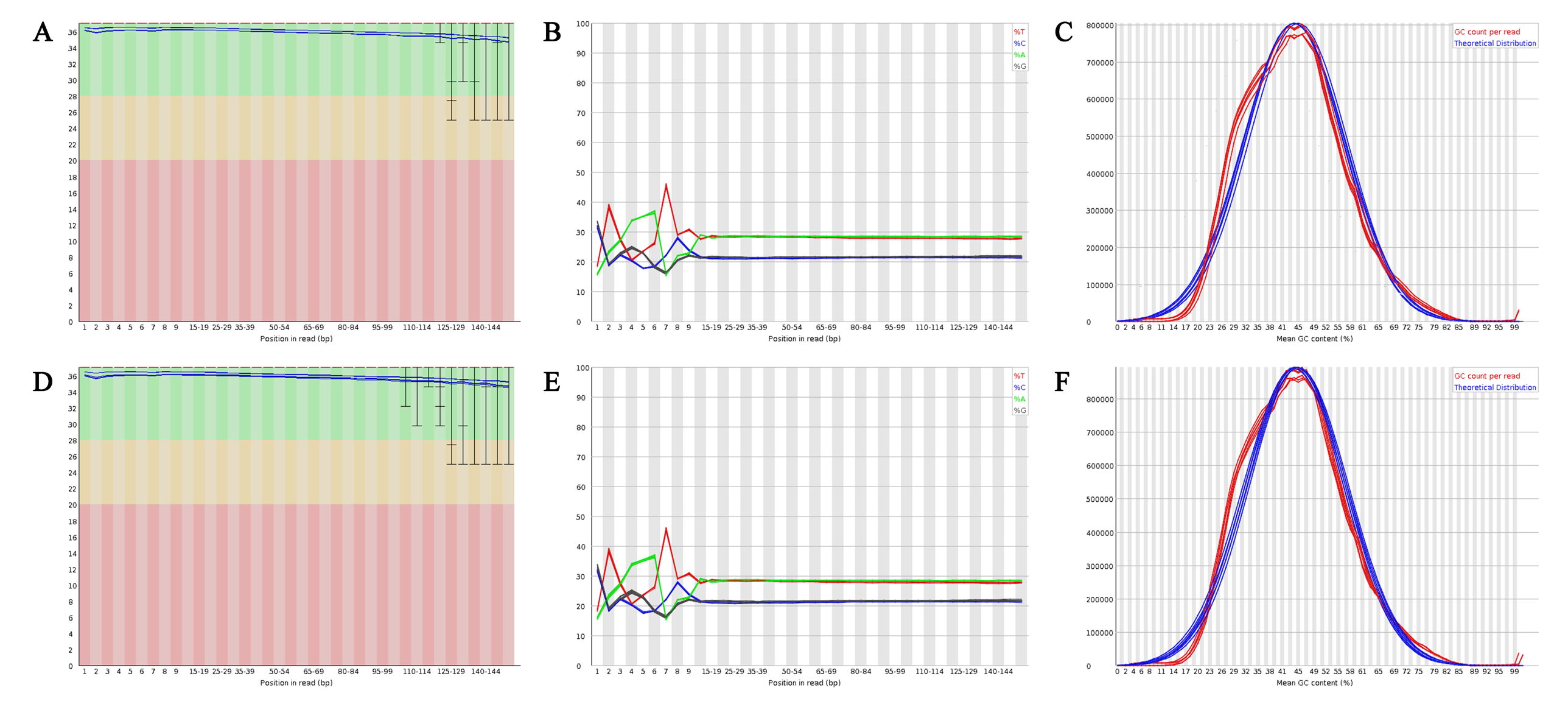

Supplement: S1 Fig — (A) Base quality value distribution in wildtype C1(H). (B) Base composition distribution in wildtype C1(H). (C) Average GC content distribution in wildtype C1(H). (D) Base quality value distribution in mutant rep-1. (E) Base composition distribution in mutant rep-1. (F) Average GC content distribution in mutant rep-1. (TIF) [file pone.0237242.s001.tif]

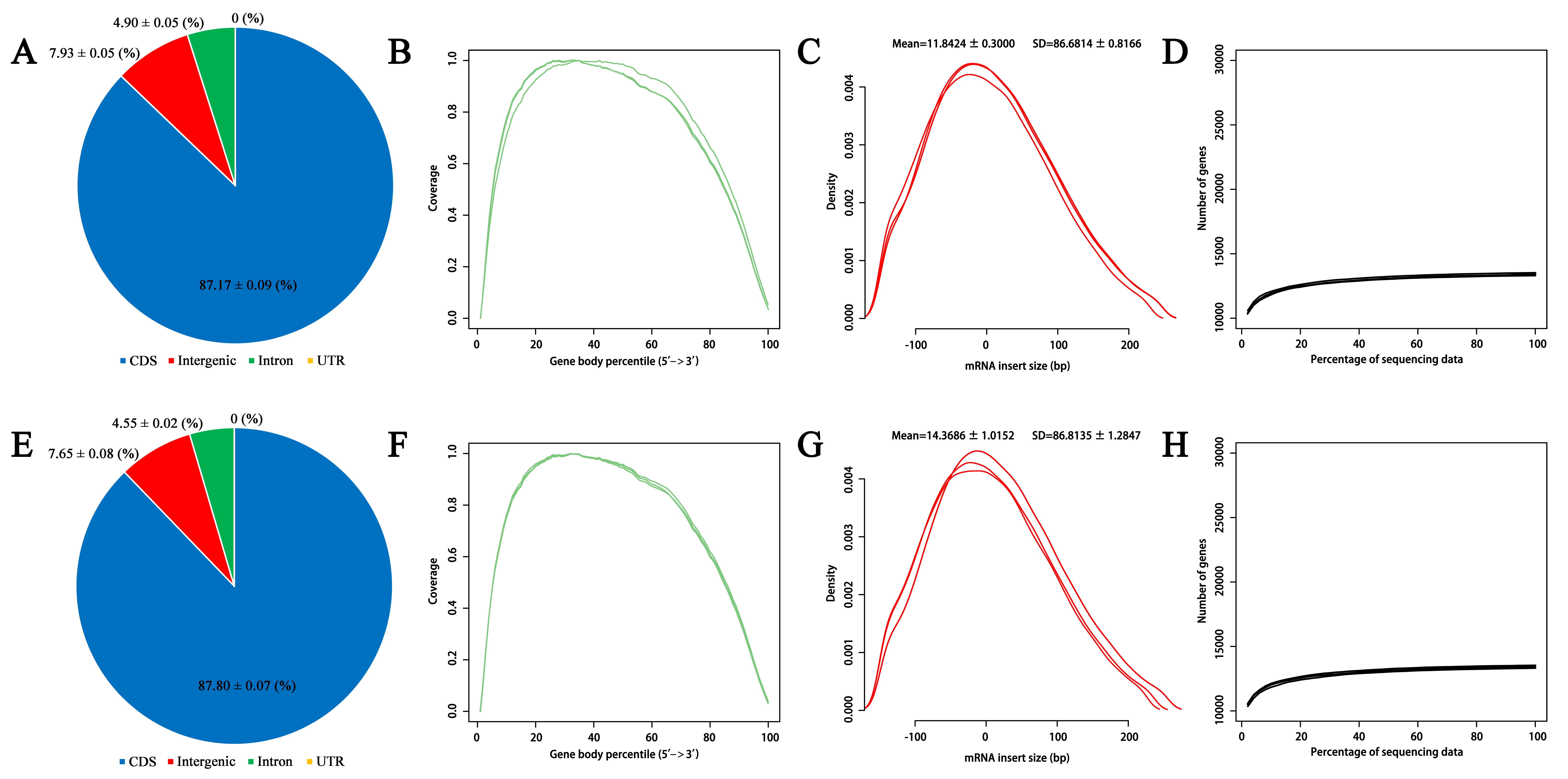

Supplement: S2 Fig — (A) Distribution of reads in different regions of genome the wild type genome. (B) Homogeneous analysis of read coverage in wild type. (C) Length analysis of insert fragment in wildtype. (D) Saturation analysis in wild type. (E) Read distribution in different regions of the mutant. (F) Homogeneous analysis of read coverage in the mutant. (G) Length analysis of insert fragments in the mutant. (H) Saturation analysis in the mutant. (TIF) [file pone.0237242.s002.tif]

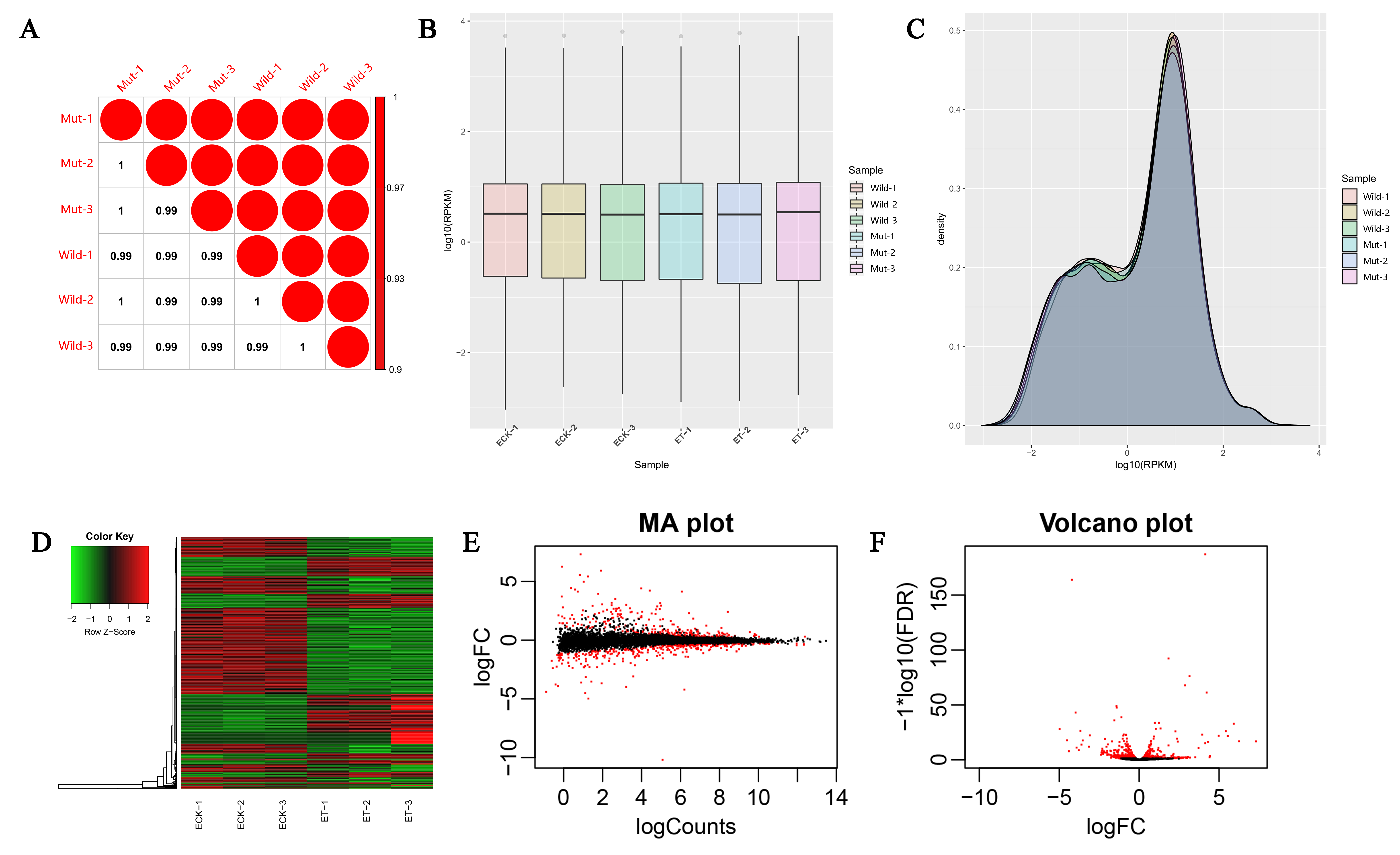

Supplement: S3 Fig — (A) Expression correlation analysis between different samples. (B) Box plots of gene expression level. (C) Density graphic of gene expression level. (D) Cluster analysis of differentially expressed genes. (E) MA plot of differentially expressed genes. (F) Volcano Plot of differentially expressed genes. (TIF) [file pone.0237242.s003.tif]
