# Supplementary material for: Transcriptome analysis of the eggs of the silkworm pale red egg (rep-1) mutant at 36 hours after oviposition
Source: PLoS One. 2020 Aug 7;15(8):e0237242. doi: 10.1371/journal.pone.0237242 (PMC7413551; doi:10.1371/journal.pone.0237242)

Manuscript Click here to access/download;Manuscript;Manuscript.doc

Title: Transcriptome analysis of the eggs of the silkworm pale red egg (*re*^{p}-1) mutant at 36 hours after oviposition

Short Title: Transcriptome analysis of the eggs of the silkworm pale red egg (*re*^{p}-1) mutant

Meina Wu[1,2], Pingyang Wang[1,2,3], Mengjie Gao[1,2], Dongxu Shen[1,2], Qiaoling Zhao[1,2]*

1 School of Biotechnology, Jiangsu University of Science and Technology, Zhenjiang, Jiangsu, 212018, China; **2** The Sericulture Research Institute, Chinese Academy of Agricultural Sciences, Zhenjiang, Jiangsu, 212018, China; **3** Guangxi Zhuang Autonomous Region Research Academy of Sericultural Science, Guangxi, Nanning, 530007, China.

* qlzhao302@126.com

Abstract

The egg stage is one of the most critical periods in the life history of silkworms, during which physiological processes such as sex determination, tissue organ formation and differentiation, diapause and pigmentation occur. In addition, egg color gradually emerges around 36 hours after oviposition. The pale red egg mutant *re*^{p}-1 was newly discovered from the C_1 (H) wild-type, which exhibits a brown egg color. In this study, the transcriptome of the eggs was analyzed 36 hours after oviposition. Between the *re*^{p}-1 mutant and the C_1 (H) wild-type, 800 differentially expressed genes (DEGs) were identified, including 325 upregulated genes and 475 downregulated genes. These DEGs were mainly involved in biological processes (metabolic process, cellular process, biological regulation and regulation of biological process and localization), cellular components (membrane, membrane part, cell, cell part and organelle) and molecular functions (binding, catalytic activity, transporter activity, structural molecule activity and molecular transducer activity). The pathway enrichment of these DEGs was performed based on the KEGG database, and the results indicated that these DEGs were mainly involved in pathways in the following categories: metabolic pathways, longevity-regulating pathway-multiple species, protein processing in endoplasmic reticulum, peroxisome, carbon metabolism and purine metabolism. Further analysis showed that a large number of silkworm growth- and development-related genes and ommochrome synthesis- and metabolism-related genes were differentially expressed, most of which were upregulated when mutated. Our research findings provide new experimental evidence for research on ommochrome pigmentation and lay the foundation for further research on the ~~formation~~ mechanism of the *re*^{p}-1 mutant.

Key words

Pale red egg mutant; ommochrome; RNA-seq; Growth and development; DEGs

1. Introduction

The silkworm (*Bombyx mori*) is an economically important insect and a fully metamorphic lepidopteran model insect [1]. The entire life cycle involves 4 stages, including eggs, larvae, pupae and moths. All diapauses of the silkworm occur in the egg stage. Therefore, the egg stage is one of the most important stages in the life cycle of the silkworm. Initially, the eggs appear light yellow, and diapause eggs gradually become gray-green, gray-purple or light brown. Other uncommon egg colors, such as red, white, brown, orange, and purple, are caused by mutations. Silkworm eggs are mainly composed of the egg shell and complex contents, including the yolk membrane, serosa, egg yolk, embryo and so on. Egg color is a comprehensive ~~expression of~~ the egg shell, serosa pigment, embryo, yolk and other colors, ~~among which~~ ommochrome in the serosa is the determining factor [2]. Ommochrome is a tryptophan metabolite in insects that prevents tryptophan poisoning [3]. Ommochrome forms different colors by participating in redox reactions and is one of the main pigment substances in insects [4]. Tryptophan is converted into formylkynurenine via the opening of the pyrrole ring under the action of tryptophan pyrrolase (TRPO), after which kynurenine formamidase (KFase) causes a change in kynurenic, which is further transformed into 3-hydroxy-canine with the aid of kynurenine 3-hydroxylase (K$_3$H) ureine. Eventually, two molecules of 3-hydroxy-kynurenine are synthesized by phenoxazinone synthetase (PHS) to produce xanthommatin [5-8]. Ommochrome is mainly found in compound eyes and eggs. Additionally, it exists in the epidermis and central nervous system of silkworm larvae [9]. 3-Hydroxykynurenine in the blood in the pupa stage enters the ovaries and compound eyes to affect egg color and adult compound eye color, respectively [10].

Egg color mutants are good study subjects for the investigation of pigment metabolism. A large number of egg color mutants are maintained in the silkworm mutant library, and many new mutants are continually being discovered. The first white egg (*w-1*) of the silkworm cannot produce ommochrome because lack of kynurenine-3-hydroxylase leads to the accumulation of kynurenine, which cannot be converted into 3-hydroxy kynurenine ~~in the body~~ [11]. The second white egg (*w-2*) of the silkworm is yellow-white in the early stage and then develops a reddish color. Researchers have shown that the mutant form results from the mutation of the ATP-binding transporter ~~via map cloning technology~~. Dimers formed by ATP-binding

cassette transporters and products encoded by the *white* gene are responsible for ommochrome transport [12]. There are many mutants involving multiple alleles in the third stage (*w-3*) in the silkworm, including the white egg worm (*w-3*^{oe}) mutant, which is caused by a single-base deletion in exon 2 of the *white3* gene [13]. The serosa cells of the red egg mutant (*re*) are red and contribute to the eggs turning dark red. Map-based cloning has proven that a new member of the transmembrane protein family causes the mutation [14].

The pale red egg mutant (pale red egg, re^p-1) is a new egg color mutant found in the silkworm breed C_1 (H). Its color gradually changes to pale red over approximately 36 hours to a color similar to that of the red egg mutant (*re*) with a different intensity (Fig 1). By hybridizing the re^p-1 mutant with normal variety p50 (orthogonal or backcross), the genetic model indicated that the mutation of pale red eggs was controlled by an autosomal recessive gene [15]. When the re^p-1 (as female) mutant crossed with the *re* mutation, or the *re* (as female) mutant crossed with the re^p-1 mutation, all eggs of F_1 were in the same color as the female parent. While the eggs ofF_2 generation were all red. But F_3 individuals appeared egg color segregation, which were in different degree of red and difficult to distinguish from red eggs and pale red eggs. Gene mapping showed that the key gene controlling re^p-1 mutant is very close in the same linkage to the *MFS* gene that controlled the *re* mutation(data not show).

Because the egg color change starts 36 hours after oviposition, to understand the gene expression profile of the mutant at this time, the eggs of the re^p-1 silkworm mutant laid within 36 hours were used as the experimental materials, and C_1 (H) as a control group. Fifty eggs of each silkworm strain were pooled for transcriptome analysis. Three experiments were repeated in each group. Differentially expressed genes (DEGs) were identified according to the sequencing results and further analyzed. At the same time, the expression levels of genes related to ommochrome synthesis and metabolism, egg development and structure and major genes of egg color mutants were analyzed to explain the possible roles of these genes in the re^p-1 pale red egg mutant. The study conduce to build a foundation for understanding the formation mechanism of re^p-1 mutants and provides a theoretical basis for the study of the molecular mechanism of egg color formation.

2. Materials and methods

2.1. The silkworm strains

The C_1 (H) and *re*^{p}-1 silkworm strains were provided by the Chinese Academy of Agricultural Sciences (Zhenjiang. China). Larvae were reared on fresh mulberry leaves in an environment with a 12-h light/12-h dark cycle at 25 ± 1℃ and 70–85% relative humidity. After 36 hours of oviposition, 50 eggs of each silkworm strain were pooled for RNA-Seq and quantitative analysis. Each assay was repeated three times. Eggs at 12h，24h and 36h after oviposition were used to quantitative analysis for ~~different genes~~. This experiment was approved by the Jiangsu University of Science and Technology Animal Care and Use Committee.

2.2. RNA extraction，library construction and RNA-Seq

RNA was extracted using RNAiso Plus (TaKaRa, China) and dissolved in RNase-free water. RNA purity was assessed by using a NanoDrop 1000 microspectro photometer (Thermo, USA). Then total mRNA treated with RNase was enriched under the action of Oligo (dT) magnetic beads. The next step was to shear mRNA into short fragments by adding hybridization interruption reagents. Double-stranded cDNA was synthesized by random six-base primers and ~~purified with~~ the interrupted mRNA as the template. The purified double-stranded cDNA was end repaired, and a tail was added and connected with a sequencing connector. PCR amplification was carried out ~~as a template~~ to generate cDNA library. The library constructed in the previous step was loaded onto the sequencing chip of Illumina HiSeq TM 2000 sequencer for sequencing (NanJing Decode Genomics). Finally, the original read data of the processing group and the control group library were obtained.

2.3. Sequence assembly

After filtering out low quality reads and sequence reads containing connectors or poly-A / T tails, clean reads were assembled by using the short reads assembler Trinity ~~software to produce unigenes~~. The unigenes from two samples were gathered together, and they were clustered by TGI clustering tool [16]. For functional annotation, ~~the~~ BLASTX was used to retrieve single gene sequence of various protein databases including Nr，Swiss-Prot，COG and KEGG [17], and ~~then use~~ BLASTN to retrieve nucleotide database ~~NT~~（E-value<10-5）.The coding region sequence (CDS) was extracted from unigenes by searching BLAST results and translated into peptide sequence. When ~~an~~ unigene does not find the matching sequence, the sequence direction was determined through estscan software. In addition, a Gene Ontology

(GO) Association was performed for Nr database according to BLASTX, and annotate each unigene with Blast2GO ~~program~~ [18,19].

2.4. Quality control of RNA-Seq data

Quality control analysis was performed on the raw data, which were filtered to obtain clean reads. The filtering rules were as follows: removal of reads with adapter sequences at first; then removal of reads with N bases (N means that the base information cannot be determined); finally removal of reads with low quality (in which the number of reads with a quality value of Qphred ≤ 5 is more than 50% of the total reads). At the same time, the distribution of base quality values, the base composition and the average GC content of the original sequence data were analyzed to ensure that the sequencing data met the requirements for subsequent analysis.

The clean reads were compared with the reference genome (NCBI, https://www.ncbi.nlm.nih.gov/), the statistical reads in the distribution of different segments of the genome and the results of the second quality analysis, including read coverage uniformity, insert sequence fragment length and sequencing saturation analyses, to ensure that the reads met the requirements of the subsequent gene expression data and DEG analyses.

2.5 Analysis of differentially expressed genes

The number of reads obtained from the genome was counted, and the RPKM (reads per kilobase per million reads) method was used to standardize the expression level of each gene. The boxplot and density distribution of the RPKM values and the correlation analysis of each sample were performed to compare the differences in gene expression levels among different samples according to the overall level of gene expression. Then, an FDR$\leq$0.05 (FDR: false discovery rate) was used as the threshold for ~~the~~ screening ~~of~~ DEGs [20]. GO and KEGG pathway enrichment analyses were performed for the differentially expressed genes encoding proteins. The expression levels of genes related to ommochrome synthesis and metabolism, genes related to egg development and structure and major genes of the egg color mutants were analyzed simultaneously to elucidate the possible roles of these genes in re^p-1 pale red egg mutants.

2.6. Quantitative reverse transcription PCR

Eggs were produced by C_1 (H) and re^p-1 mutant silkworms for 36 hours. For each sample, 50 eggs were pooled. The RNAiso plus method was used to extract the

total RNA, followed by treatment with DNase, and cDNA was obtained using an M-MLV Reverse Transcriptase (RNase H-) kit. The cDNA was diluted to 100 ng/μl as a template for qRT-PCR. All qRT-PCR reactions were performed in a 20 μl volume containing 1μl specific primers (10 μ[illegible]), 1μl cDNA, 10 μl NovoStart®SYBR qPCR SuperMix (Novoprotein, Nanjing, China) and H_2O in a LightCycler® 96 quantitative PCR instrument. The reaction conditions were as follows: predenaturation at 95°C for 10 min, 40 three-step cycles including denaturation at 95°C for 10 s, annealing at 58°C for 10 s, and extension at 72°C for 10 s(58°C to 95°C in an increment of 0.5 °C every 5 s). Finally, the melting curve was generated. The relative expression of each gene was calculated in relation to the average values for two housekeeping genes, RPL-3 (*BGIBMGA013567*) and GAPDH (*BGIBMGA007490*). The $2^{-\Delta\Delta Ct}$ method [21] was used to analyze the melting curves of each gene to verify the high efficiency and ~~product specificity of PCR amplification~~. qRT-PCR expression in the wild-type was compared with ~~that in~~ *re*^{p}-1 to verify the accuracy of the DEG data.

3 Results

3.1. General information for RNA-Seq

Eggs that hatched after 36 hours were used for RNA-seq. Mega raw reads were obtained from the wild-type C_1 (H) group (55.72 ± 6.22), and Mega clean reads (55.54 ± 6.19) were obtained by removing unqualified reads through quality control. Similarly, Mega raw reads (64.08 ± 7.50) and Mega clean reads (63.88 ± 7.50) were obtained from the *re*^{p}-1 mutant. The ratios of clean reads and raw reads reached 99.67% and 99.68%, respectively. The GC content of the reads for both the wild-type C_1 (H) group and the *re*^{p}-1 mutant was approximately 43%, and that of adapter reads was approximately 3% (Table 1).

The analysis of distribution of the base mass values of the sequencing data (S1_Fig 1A.1D), the distribution of the composition of each base (S1_Fig 1B.1E) and the distribution of the average GC content (S1_Fig 1C.1F) showed that the base mass was good; the base composition was uniform; and the GC content was stable, thus satisfying the requirements for subsequent analysis. In addition, the Q20 values of the raw reads and clean reads of C_1 (H) and *re*^{p}-1 were greater than 95%. The Q30 values of the raw reads and clean reads of C_1 (H) and *re*^{p}-1 were greater than 90% (Table 1), thus meeting the requirements for subsequent analysis.

3.2. Read mapping analysis

The clean reads obtained from RNA-seq were compared with the NCBI reference genome. In C_1 (H) and *re*^{p}-1, 87.09% and 87.46% of the reads were compared to the reference genome. A total of 83.44% and 83.90% of the reads, respectively, were compared to the single-copy reference sequence, and 2.72% and 2.69% of the reads were compared to the multicopy reference sequence. The number of clean reads exceeded 55 mega, reaching the minimum requirement of 10 mega, and the unique gene mapping ratio reached 80%.

The distribution statistics of the reads in different regions of the genome showed that more than 87% of the reads were matched to CDSs, and some of the reads were matched to intron and gene intervals, possibly due to the presence of unknown genes and alternative splicing (S2_Fig 2A.2E) The coverage uniformity analysis of the reads showed that the coverage of 10 windows at the 5' end was limited. The coverage of 10 to 60 windows was best. The coverage of the 3' end gradually decreased (S2_Fig 2B. 2F). The analysis of insert length showed that the average distances between two reads in C_1 (H) and *re*^{p}-1 were 11 BP and 14 BP, respectively, which showed that the selected length could basically cover two reads and achieved the maximum utilization of the sequence (S2_Fig 2C.2G). The saturation analysis indicated that saturation was reached when the sequencing percentage was approximately 40%, and all of the sequencing requirements were met (S2_Fig 2D) Therefore, the above analysis of the mapping results showed quite good results and met the requirements for subsequent analysis.

3.3. General gene expression information

In total, we analyzed the expression levels of 15884 gene in *Bombyx mori* (S2_Table). Among these genes, 12403 were expressed in wild-type C_1 (H), accounting for 78.08% of the total, while 12488 (78.62%) were expressed in the *re*^{p}-1 mutant (3 replicates could be detected). A total of 12007 (75.60%) genes were expressed in both C_1 (H) and *re*^{p}-1 (6 replicates could be detected); 396 (2.50%) genes were expressed in C_1 (H) but not in *re*^{p}-1; and 481 genes were expressed in *re*^{p}-1 but not in C_1 (H), accounting for 3.03% of the total. Finally, 3000 genes were not expressed in C_1 (H) and *re*^{p}-1 (could not be detected in at least one of the six samples), accounting for 18.89% of the total (Table 1).

The correlation analysis results for sample expression showed that the correlations between the six samples were very close; the correlation coefficient was close to 1 (S3_Fig 3A); the box plot analysis results showed that the repeatability of

the gene expression results of the six samples was good. The maximum value, the upper quartile, the median value, the lower quartile and the minimum value showed good uniformity among the six samples (S3_Fig 3B). A horizontal density distribution diagram of gene expression was provided. Genes showing an expression value of approximately 10 were the most abundant (the first peak). Genes with an expression value of approximately 0.1 were also highly expressed (the second peak) (S3_Fig 3C).

3.4. Analysis of differentially expressed genes

An FDR $\leq$ 0.05 was used as the screening criterion for the screening of differentially expressed genes from S3_Table We ~~have~~ identified 800 differentially expressed genes (S3_Table). Compared with the C_1 (H) wild-type, 475 genes in the *re*^{p}-1 mutant were downregulated, and 325 genes were upregulated (S3_Fig 3D-3F). Go cluster analysis was carried out for these differentially expressed genes. Among these genes, the identified biological processes mainly included the following terms: metabolic process (103 upregulated genes and 115 downregulated genes), cellular process (85 up and 115 down), biological regulation (27 up and 66 down), regulation of biological process (24 up and 59 down) and localization (22 up and 46 down). The identified cellular component terms mainly included the following: membrane (67 up and 110 down), membrane part (60 up and 96 down), cell (62 up and 80 down), cell part (61 up and 80 down) and organelle (41 up and 35 down). The molecular function terms were mainly related to binding (100 up and 142 down), catalytic activity (104 up and 133 down), transporter activity (8 up and 28 down), structural molecule activity (6 up and 7 down) and molecular transducer activity (3 up and 12 down) (Fig 2. S4_Table), Take the difference multiple greater than 2, that was |log2(FOLD change) |>1, 114 genes were upregulated and 120 were downregulated, and the 234 differentially expressed genes were mainly related to binding proteins (56) and catalytic activity (64).

These differentially expressed genes were analyzed by using the KEGG database to evaluate pathways, and 218 genes were clustered into 105 pathways. The top 6 clustered genes belonged to the following categories: metabolic pathways (89), longevity regulating pathway - multiple species (16), protein processing in the endoplasmic reticulum (16), peroxisome (14), carbon metabolism (14) and purine metabolism (14) (Fig 3. S5_Table). Among the metabolic pathways, a large number of amino acids metabolic pathways showed changed, including those of serine, cysteine,

methionine, leucine, lysine, arginine, proline, tyrosine, polyamines, p-aminobutyric acid and glutathione.

3.5 Verifying the accuracy of the RNA-Seq data by qRT-PCR

To verify the accuracy of the RNA-seq data, we analyzed the expression of three housekeeping genes, and selected certain genes for qRT-PCR testing, including some differentially expressed genes, pigment synthesis genes, metabolism-related genes, egg shell protein genes, yolk membrane protein genes, and major genes of egg color mutants (Table 2. S1_Table).

Three housekeeping genes, *bmactin3* (fold-change=1.07). Glyceraldehyde-3-phosphate dehydrogenase (fold-change=0.81) and ribosomal protein L3 (fold-change=0.97), showed fold-changes in all databases that were approximately equal to 1, providing initial verification that the RNA-seq data were accurate. Twelve upregulated genes and 12 downregulated genes with fold-change differences greater than 2 were randomly selected for qRT-PCR validation. The results for 20 out of 24 DEGs were identical to the qRT-PCR results, whereas the qRT-PCR results for 2 out of 4 genes were inconsistent with the DEG results. However, the fold-change difference was not significant (0.5 < fold-change < 2). Therefore, the results for 22 of the DEGs genes were consistent with the qRT-PCR results. The accuracy was 91.67%, which indicated that the DEG results were reliable. The expression levels of some genes are shown in Fig 4.

4. Discussion

4.1. Differentially expressed genes based on RNA-Seq data

Serotonin shows a widespread distribution in the insect nervous system[22]. It affects sensory processing, information coding and behavior [23]. The glutamate-gat chloride channel protein is only found in invertebrates, mediating fast inhibitory neurotransmission [24]. The genes encoding these two proteins involved in the nervous system were downregulated (fold-change=0.49) and significantly upregulated (fold-change=11.69), respectively, suggesting that the development of the nervous system might be affected in the mutant. Troponin plays a role in muscle contraction [25]. Dynein plays a role in the movement of flagella and cilia [26]. The genes encoding these two proteins are related to motility and were upregulated in the mutant (fold-change=3.71 for troponin, while dynein was expressed only in the mutant). These results indicate that material transport and metabolism in the mutant are

relatively similar. Other upregulated genes among the genes involved in the metabolism of these substances included those encoding ~~and~~ a key enzyme in the urea cycle, arginine succinate lyase, was also upregulated (argininosuccinate lyase, fold-change = 16.83) [27], the Krebs cycle (tricarboxylic acid cycle) component isocitrate dehydrogenase (isocitrate dehydrogenase, fold-change = 3.28) [28], a protein with a role in cell adhesion (cytoadherence) (integrin, fold-change [29] = 7.48), the signal transduction-related enzyme serine-threonine protein kinase (serine/threonine - protein kinase, fold-change = 4.89) [30, 31] and the acyl-phosphate dependent sn-glycerol-3-phosphate acyltransferase (old-change=26.66) involved in glycerolipid synthesis [32], further confirming the similar cellular activity and material metabolism of the mutant.

4.2. Differentially expressed genes involved in amino acid metabolism

KEGG pathway clustering analysis of the differentially expressed genes revealed 89 DEGs clustering to metabolic pathways. The analysis revealed many amino acid metabolic pathways showing gene expression differences (S5_Table), including the serine, betaine, cysteine, arginine, proline, polyamine and glutathione biosynthesis pathways, methionine, leucine, lysine and tyrosine degradation pathways and aminobutyric acid metabolic pathways. Amino acids are not only the basic substances necessary for biological activities but are also the components of basic molecules such as proteins, which are involved in almost all physiological processes. Abnormal metabolism will affect the growth and development of the silkworm.

4.3. Differentially expressed genes related to ommochrome synthesis

Ommochrome is the key substance responsible for the formation of egg color. 3-Hydroxykynurenine, which is synthesized from tryptophan through a series of catalytic reactions, is transported to pigment particles by the ABC transporter heterodimer. Then, it is transformed via oxidative condensation to form purple ommin pigment and red-brown xanthommatin pigment [10] (Fig 5). In silkworms the scarlet protein encoded by the *Bmw*-2 gene and the white protein encoded by the *Bmwh3* gene form a white/scarlet dimer, which transports 3-hydroxykynurenine, an ommochrome precursor, to pigment granules [12, 13]. The *Bmcardinal* gene encodes phenoxazinone synthase, which catalyzes the synthesis of xanthommatin. The other 3-hydroxykynurenine is used to synthesize ommin, which plays a key role in red egg mutants. The mutant gene MFS is related to the formation of ommin [14]. Kynurenine 3-hydroxykynurenine can simultaneously be hydrolyzed to produce anthranilic acid

and 3-hydroxy-2-aminobenzoic acid via the action of the kynureninase gene *bmkynu* [33] (Fig 5).

The quantitative analysis of gene expression in the ommochrome synthesis and metabolism pathways at 12 hours, 24 hours and 36 hours after oviposition showed that the expression levels of most genes were upregulated in the re^{p}-1 mutant. Compared to the C_1 (H) wild-type, there was a trend of increasing expression levels in eggs in the 12 hours postpartum period. Expression was significantly increased at 24 hours and tended to return to a level similar to that in the wild-type at 36 hours (Fig 5). According to the analysis of gene expression, the main effector gene of the re^{p}-1 mutant caused changes in gene expression in the ommochrome synthesis pathway. A series of genes were upregulated in the mutant, causing more ommochrome to be produced. In relation to ovary coloration and the phenotypic changes in mutant pale red eggs, the changes in gene expression at different developmental stages were analyzed. The genes related to egg pigment synthesis began to be expressed at high levels at 12 hours after oviposition, reaching their highest expression at 24 hours, after which their expression began to decrease. At 36 hours, ommochrome synthesis tended to be complete. Egg color began to appear, and gene expression was reduced to the lowest level. In particular, the expression levels of the two ABC transporters responsible for transporting 3-hydroxykynurenine to pigment granules reached a maximum at 24 hours. The results showed that 24 hours after ovipositionmay be the key period of ommochrome synthesis in silkworm eggs.

4.4 Expression analysis of key genes of several egg color mutants

Silkworm eggs represent not only the end of life but also the beginning of a new cycle. Egg diapause is useful for surviving in harsh environments, and the egg stage is therefore a very important developmental period. Egg color is a critical physiological characteristic of silkworm eggs. Normal silkworm eggs are gray-green, gray-purple or light brown. Many different egg color mutants have been identified in the silkworm breeding. These egg color mutants are essential for studying silkworm egg pigment deposition. The color mutations and mutant genes identified thus far are shown in Table 2.

The mutant egg color genes that were identified were all related to the ommochrome synthesis pathway. Quantitative analysis showed that these genes were upregulated in the pale red egg mutant. Most of the genes were upregulated at 24 hours after oviposition (Fig 5). The results indicated that the egg color mutants largely

result from mutations in the ommochrome synthesis pathway. The white egg mutant is unable to complete ommochrome synthesis because of gene mutations during the early stage of the synthesis pathway. However, compared to the white egg mutant, egg color mutations are caused by later gene mutations that result in different egg color phenotypes according to different ratios of the mixture of ommin (purple) and xanthommatin (red-brown).

4.5. Differentially expressed genes encoding structural proteins of eggs

Insect eggs are mainly composed of the shell and its contents, including the yolk membrane, protoplasm, vitellus, cell nucleus and other structures. The membrane protoplasm as noncellular structure is located close to the egg shell. The vitellus s very important because of its complex components, which provide nutrition for the development of the egg. The nucleus is responsible for the storage of genetic information. The egg shell is a complex protective structure on the surface of the egg with the physiological function of protecting embryos, gas exchange and sperm in the eggs [34]. The egg shell protein s the main component of the egg shell. A total of 96% of the dry weight of the silkworm, is contributed by the egg shell protein. More than 100 egg shell proteins have been identified in silkworms to date [35, 36]. According to RNA-Seq data analysis, 8 chorion-related genes were expressed. However, there was no significant difference in the expression of these genes (|log2(fold-change)|<0.5). The results showed that egg shell construction had been completed 36 hours after oviposition. Only a few egg shell proteins were expressed. The mutant genes of the pale red egg mutant did not affect the regulation and expression of egg shell proteins (S6_Table). The yolk protein precursor is mainly derived from the female-specific blood protein vitellogenin (Vg) of the adipose body. After synthesis by the adipose body, vitellogenin is transferred to the ovary through the hemolymph, which is absorbed by oocytes and deposits vitellogenin in the egg to provide nutrients and functional substances for embryonic development [37-39]. By analyzing the expression of the vitellogenin gene, we found that the expression of all vitellogenin genes was upregulated in the light red egg mutants except for one gene. The skin an

essential organ for the avoidance of harm, growth reproduction and adaptation to complex environments in insects [40, 41]. In the insect genome, there are many different structural characteristics of the cuticular protein genes (CPGs). There are more than 200 genes encoding CPGs in the silkworm genome [40, 42]. Approximately 30 CPGs were expressed in the 36 hours ~~-instar~~ silkworm eggs. However, there was no significant difference between the wild-type and the mutants. In conclusion, the expression of genes encoding silkworm egg structural proteins was not significantly affected apart from some genes encoding vitellogenin.

Conclusion

In this study, the silkworm eggs of the light red pale egg mutant re^{p}-1 at 36 hours after ~~the~~ oviposition were used as the experimental group, and those of the C_1 (H) wild-type were used as the control group for RNA-seq data analysis. The qRT-PCR results verified the accuracy of the RNA-seq data. A total of 800 DEGs were identified by RNA-seq. Compared with C_1 (H), 475 genes were downregulated and 325 genes were upregulated in re^{p}-1. It was found that the expression of genes related to the growth and development of silkworms and ommochrome synthesis and metabolism ~~was~~ changed significantly. The overall trend was that the expression level was upregulated in the mutants. At the same time, numerous DEGs related to ~~the~~ amino acid metabolism pathway were found.

Supporting Information

S1_Fig. Quality control of sequencing base.

(A) Base quality value distribution in wildtype C_1(H). (B) Base composition distribution in wildtype C_1(H). (C) Average GC content distribution in wildtype C_1(H). (D) Base quality value distribution in mutant re^{p}-1. (E) Base composition distribution in mutant re^{p}-1. (F) Average GC content distribution in mutant re^{p}-1.

S2_Fig. Quality control of genome alignment.

(A) Reads distribution in different regions of genome in wildtype. (B) Homogeneous analysis of read~~s~~ coverage in wildtype. (C) Length analysis of insert fragment in wildtype. (D) Saturation analysis in wildtype. (E) Read~~s~~ distribution in different regions of genome in mutant. (F) Homogeneous analysis of read~~s~~ coverage in mutant.

(G) Length analysis of insert fragment in mutant. (H) Saturation analysis in mutant.

S3_Fig. Analysis of general expression and differentially expressed genes. (A) Expression correlation analysis between different samples. (B) Box plots of gene expression level. (C) Density graphic of gene expression level. (D) Cluster analysis of differentially expressed genes. (E) MA plot of differentially expressed genes. (F) Volcano Plot of differentially expressed genes.

S1_Table Primers. Expression levels and descriptions of differentially expressed genes.
(xlsx)

S2_Table Reads number and RPKM values of all genes.
(xlsx)

S3_Table Differentially expressed genes.
(xlsx)

S4_Table Gene ontology analysis of differentially expressed genes.
(xlsx)

S5_Table KEGG pathway enrichment.
(xlsx)

S6_Table Genes encoding chorion. Cpgs. Vitellogenin and ommochrome synthesis related enzymes.
(xlsx)

Acknowledgement

This work was supported by grants from the National Natural Science Foundation of China (No. 31972616) and the Innovation Plan of Jiangsu Province (KYCX19_1656). We are very grateful to Simin Bi and Chenjie Yang for their help and assistance in this study.

References

1. Qin J, He N, Xiang Z. Advances in silkworm modeling research. Science of Sericulture. 2010; 36(4): 645-649.
2. Zhang H, Lin Y, Shen G, Tan X, Lei C, Long W, Liu H, Zhang Y, Xu Y, Wu J, Gu J, Xia Q, Zhao P. Pigmentary analysis of eggs of the silkworm *Bombyx mori*. J Insect Physiol. 2017; 101: 142-150.
3. Insausti TC, Casas J. The functional morphology of color changing in a spider: development of ommochrome pigment granules. J Exp Biol. 2008; 211(Pt 5): 780-789.
4. Linzen B. The tryptophan omrnochrome pathway in insects. Adv Insect Physiol. 1974; 10: 117-246.
5. Hayashi Y. Properties of xanthine dehydrogenase in the silkworm, *Bombyx mori* L. Nature. 1961; 192: 756-757.
6. Phillips JP, Forrest HS. Ommochromes and pteridines. In: Ashburmer M, Wright TRF, editors. The Genetics and Biology of *Drosophila*. London, UK: Academic Press. 1980. pp. 542–623.
7. Wang P, Qiu Z, Xia D, Tang S, Shen X, Zhao Q. Transcriptome analysis of the epidermis of the purple quail-like (*q-l*^{p}) mutant of silkworm, *Bombyx mori*. PLoS One. 2017; 12(4): e0175994.
8. Osanai-Futahashi M, Tatematsu KI, Futahashi R, Narukawa J, Takasu Y, Kayukawa T, Shinoda T, Ishige T, Yajima S, Tamura T, Yamamoto K, Sezutsu H. Positional cloning of a *Bombyx* pink-eyed white egg locus reveals the major role of cardinal in ommochrome synthesis. Heredity (Edinb). 2016; 116(2): 135-145.
9. Sawada H, Nakagoshi M, Mase K, Yamamoto T. Occurrence of ommochrome-containing pigment granules in the central nervous system of the silkworm, *Bombyx mori*. Comp Biochem Physiol B Biochem Mol Biol. 2000; 125(3): 421-428.
10. Peng C, Wu S, Dai F. Progress of molecular genetic studies on pigmentation pattern in silkworm, *Bombyx mori*. Science of Sericulture. 2016; 42(3): 373-385.
11. Quan GX, Kim I, Komoto N, Sezutsu H, Ote M, Shimada T, Kanda T, Mita K, Kobayashi M, Tamura T.Characterization of the kynurenine 3-monooxygenase gene corresponding to the white egg 1 mutant in the silkworm *Bombyx mori*. Mol Genet Genomics. 2002; 267(1): 1-9.
12. Tatematsu K, Yamamoto K, Uchino K, Narukawa J, Iizuka T, Banno Y, Katsuma S, Shimada T, Tamura T, Sezutsu H, Daimon T. Positional cloning of silkworm white egg 2 (*w-2*) locus shows functional conservation and diversification of ABC transporters for pigmentation in insects. Genes Cells. 2011; 16(4): 331-342.
13. Komoto N, Quan GX, Sezutsu H, Tamura T. A single-base deletion in an ABC transporter gene causes white eyes, white eggs and translucent larval skin in the silkworm w-3 (oe) mutant. Insect Biochem Mol Biol. 2009; 39(2): 152-156.
14. Osanai-Futahashi M, Tatematsu K, Yamamoto K, Narukawa J, Uchino K, Kayukawa T, Banno Y, Tamura T, Sezutsu H. Identification of the *Bombyx* red egg gene reveals involvement of a novel transporter family gene in late steps of the insect ommochrome biosynthesis pathway. J Biol Chem. 2012; 287(21): 17706-17714.
15. He F, Zhao Q, Wang M, Wang P, Xia D, Qiu Z, Tang S, Sheng X. Discovery of the pale red egg (*re*^{p}-1) mutant in *Bombyx mori* and structure analysis of *mfs* gene related to egg color. Science of

Sericulture. 2017; 43(6): 913-921.

16. Pertea G, Huang X, Liang F, Antonescu V, Sultana R, Karamycheva S, Lee Y, White J, Cheung F, Parvizi B, Tsai J, Quackenbush J. TIGR Gene Indices clustering tools (TGICL): a software system for fast clustering of large EST datasets. Bioinformatics. 2003; 19(5): 651-652.
17. F. Altschul S, L. Madden T, A. Schäffer A, Zhang J, Zhang Z, Miller W, J. Lipman D. Gapped BLAST and PSI-BLAST: a new generation of protein database search programs. Nucleic Acids Res. 1997; 25(17): 3389–3402.
18. Conesa A, Götz S, García-Gómez JM, Terol J, Talón M, Robles M. Blast2GO: a universal tool for annotation, visualization and analysis in functional genomics research. Bioinformatics. 2005; 21(18): 3674–3676.
19. Gotz S, Garcia-Gomez JM, Terol J, Williams TD, Nagaraj SH, Nueda MJ, Robles M, Talon M, Dopazo J, Conesa A. High-throughput functional annotation and data mining with the Blast2GO suite. Nucleic Acids Res. 2008; 36(10): 3420–3435.
20. Benjamini Y, Yekutieli D. The control of the false discovery rate in multiple testing under dependency. Annals of Statistics. 2001; 29(4): 1165-1188.
21. Livak KJ, Schmittgen TD. Analysis of relative gene expression data using real-time quantitative PCR and the 2 (-Delta Delta C(T)) method. Methods. 2001; 25(4): 402-408.
22. Xiang Z, Huang J, Xia J, Lu C. Biology of Sericulture. China Forestry Publishing House (CFPH), Beijing. 2005, 1–28.
23. Dacks AM, Dacks JB, Christensen TA, Nighorn AJ. The cloning of one putative octopamine receptor and two putative serotonin receptors from the tobacco hawkmoth. *Manduca sexta*. Insect Biochem Mol Biol. 2006; 36(9): 741-747.
24. Wang X, O´Reilly AO, Williamson MS, Puinean AM, Yang Y, Wu S, Wu Y. Function and pharmacology of glutamate-gated chloride channel exon 9 splice variants from the diamondback moth *Plutella xylostella*. Insect Biochem Mol Biol. 2019; 104: 58-64.
25. Chen JQ, Chen J, Gai QJ, Lv ZB, Wang D, Nie ZM, et al. Molecular characterization and immunohistochemical localization of a novel troponin C during silkworm development. Cell Tissue Res. 2008; 331(3): 725-738.
26. Mohri H, Inaba K, Ishijima S, Baba SA. Tubulin-dynein system in flagellar and ciliary movement. Proc Jpn Acad Ser B Phys Biol Sci. 2012; 88(8): 397-415.
27. Nagamani SC, Erez A, Lee B. Argininosuccinate lyase deficiency. Genet Med. 2012; 14(5): 501-507.
28. Kanao T, Kawamura M, Fukui T, Atomi H, Imanaka T. Characterization of isocitrate dehydrogenase from the green sulfur bacterium *Chlorobium limicola*: A carbon dioxide-fixing enzyme in the reductive tricarboxylic acid cycle. Eur J Biochem. 2002; 269(7): 1926-1931.
28. Davis SP, Lee K, Gillrie MR, Roa L, Amrein M, Ho M. CD36 recruits alpha (5) beta (1) integrin to promote cytoadherence of *P. falciparum*-infected erythrocytes. PLoS Pathog. 2013; 9(8): e1003590.
30. Van Lint J, Agostinis P, Vandevoorde V, Haegeman G, Fiers W, Merlevede W, et al. Tumor necrosis factor stimulates multiple serine/threonine protein kinases in Swiss 3T3 and L929 cells:

implication of casein kinase-2 and extracellular signal-regulated kinases in the tumor necrosis factor signal transduction pathway. J Biol Chem. 1992; 267(36): 25916-25921.

31. Rapp UR. Role of Raf-1 serine/threonine protein kinase in growth factor signal transduction. Oncogene. 1991; 6(4): 495-500.
32. Hara Y, Seki M, Matsuoka S, Hara H, Yamashita A, Matsumoto K. Involvement of PlsX and the acyl-phosphate dependent sn-glycerol-3-phosphate acyltransferase PlsY in the initial stage of glycerolipid synthesis in *Bacillus subtilis*. Genes Genet Syst. 2008; 83(6): 433-442.
33. Meng Y, Katsuma S, Mita K, Shimada T. Abnormal red body coloration of the silkworm, *Bombyx mori*, is caused by a mutation in a novel kynureninase. Genes Cells. 2009; 14(2): 129-140.
34. Regier JC, Mazur GD, Kafatos FC. The silkmoth chorion: morphological and biochemical characterization of four surface regions. Dev Biol. 1980; 76(2): 286-304.
35. Regier JC, Mazur GD, Kafatos FC, Paul M. Morphogenesis of silkmoth chorion: initial framework formation and its relation to synthesis of specific proteins. Dev Biol. 1982; 92(1): 159-174.
36. Chen Z, Nohata J, Guo H, Li S, Liu J, Guo Y, et al. A comprehensive analysis of the chorion locus in silkmoth. Sci Rep. 2015; 5: 16424.
37. Falchuk KH, Montorzi M. Zinc physiology and biochemistry in oocytes and embryos. Biometals. 2001; 14(3-4): 385-395.
38. Byrne BM, Gruber M, Ab G. The evolution of egg yolk proteins. Prog Biophys Mol Bio. 1989; 53(1): 33-69.
39. Lin Y, Meng Y, Wang YX, Luo J, Katsuma S, Yang CW, et al. Vitellogenin receptor mutation leads to the oogenesis mutant phenotype "scanty vitellin" of the silkworm, *Bombyx mori*. J Biol Chem. 2013; 288(19): 13345-13355.
40. Futahashi R, Okamoto S, Kawasaki H, Zhong YS, Iwanaga M, Mita K, et al. Genome-wide identification of cuticular protein genes in the silkworm, *Bombyx mori*. Insect Biochem Mol Biol. 2008; 38(12): 1138-1146.
41. Busse MS, Arnold CP, Towb P, Katrivesis J, Wasserman SA. A kappaB sequence code for pathway-specific innate immune responses. Embo Journal. 2007; 26(16): 3826-3835.
42. Liang J, Zhang L, Xiang Z, He N. Expression profile of cuticular genes of silkworm, *Bombyx mori*. BMC Genomics. 2010; 11(1): 1702-1716.

Figure Legends

Fig 1. Comparison of silkworm egg color.

[illegible]Normal type C_1(H) .(B) Red egg (*re*) mutant. (C) Pale red egg (*re*^{p}-1) mutant.（D）Normal type C_1(H) after 36 hours of oviposition . (E) Pale red egg (*re*^{p}-1) mutant after 36 hours of oviposition .

Finally, The eggs of normal type C_1(H) was black colors. the eggs of *re*^{p}-1 mutant was pale red colors, the eggs of *re* mutant was red colors

Fig 2. Gene ontology enrichment of differentially expressed genes.

These DEGs were mainly involved in biological process, cellular component and molecular function.

Fig 3. Scatter plot of KEGG pathway enrichment.

Rich factor is the ratio of differentially expressed gene numbers annotated in this pathway term to all gene numbers annotated in this pathway term. Greater rich factor means greater inte[illegible]veness.

Fig 4. Relative expression of partial genes verified by qRT-PCR.

The [illegible]nber in title represented the gene id in NCBI. All genes were closely consistent between RNA-seq and qRT-PCR, which indicated that the ~~sequencing~~ data ~~of RNA-seq~~ was believable.

Fig 5. Ommochrome synthesis pathway.

Ommochrome was synthesized through a series of enzyme catalysis employing tryptophan as the substrate. Most genes encoding these enzymes were up-regulated in *re*^{p}-1 mutant especially in 24 hours ~~ours~~ after oviposition. The ~~label for~~ vertical axis was a ratio ~~(~~expression of *re*^{p}-1 mutant / expression of wildtype~~)~~.If the ratio> 2 or <0.5, It indicates that there are differences in expression quantity.

Table 1 General information of RNA-seq data

**Category**	**Parameter**	**Wildtype**	***re*^{p}-1 mutant**
Raw data	Total Reads (Mega)	55.72 ± 6.22	64.08 ± 7.50
	Total bases (Gig[illegible]bit)	8.35 ± 0.92	9.61 ± 1.13
	Q20 (%)	97.56 ± 0.05	97.41 ± 0.06
	Q30 (%)	93.38 ± 0.11	93.08 ± 0.13
	Adapter reads (%)	3.13 ± 0.09	3.03 ± 0.09
	GC (%)	43.33 ± 0.09	43.32 ± 0.20
Clean data	Total Reads (Mega)	55.54 ± 6.19	63.88 ± 7.50
	Total bases (Gigabit)	8.31 ± 0.92	9.56 ± 1.12
	Q20 (%)	97.76 ± 0.05	97.62 ± 0.05
	Q30 (%)	93.61 ± 0.10	93.31 ± 0.12
	GC (%)	43.27 ± 0.09	43.27 ± 0.20
	Clean data/Raw data (%)	99.67	99.68
Quality Control	Clean Read Q20(%) ≥ 95 (Y or N)	Y	Y
	Clean Read Q30(%) ≥ 90 (Y or N)	Y	Y
	Clean Reads ≥ 10M (Y or N)	Y	Y
	Gene Unique Mapping Ratio(%) ≥ 80 (Y or N)	Y	Y
Genome-Mapping	Total Mapped Reads (%)	87.09 ± 0.01	87.46 ± 0.01
	Unique Match (%)	83.44 ± 0.01	83.90 ± 0.01
	Multi-position Match (%)	2.72 ± 0.01	2.69 ± 0.01
	Total Unmapped Reads (%)	12.91 ± 0.01	12.54 ± 0.01
Gene expression	Total Genes	15884	15884
	Expressed genes (%)	12403 (78.08%)	12488 (78.62%)
	Both expressed genes (%)	12007 (75.59%)	
	Not expressed genes (%)	3481 (21.91%)	3396 (21.38%)
	Neither expressed genes (%)	3000 (18.89%)	
	Expressed only in C1(H) (%)	396 (2.49%)	—
	Expressed only in *re*^{p}-1 (%)	—	481 (3.03%)

Table 2. Several egg color mutants

**Mutant**	**Mutant gene**	**Gene products**	**References**
pe	*Bmcardinal*	Phenoxazinone synthase	[8]
re	*Bmre*	MFS transporter	[14]
w-1	*BmK3H*	Kynurenine 3-hydroxylase	[11]
w-2	*Bmw-2*	Scarlet (ABC transporter)	[12]
w-3^{oe}	*Bmwh3*	White (ABC transporter)	[13]

Click here to access/download;Figure;Fig 1.tif

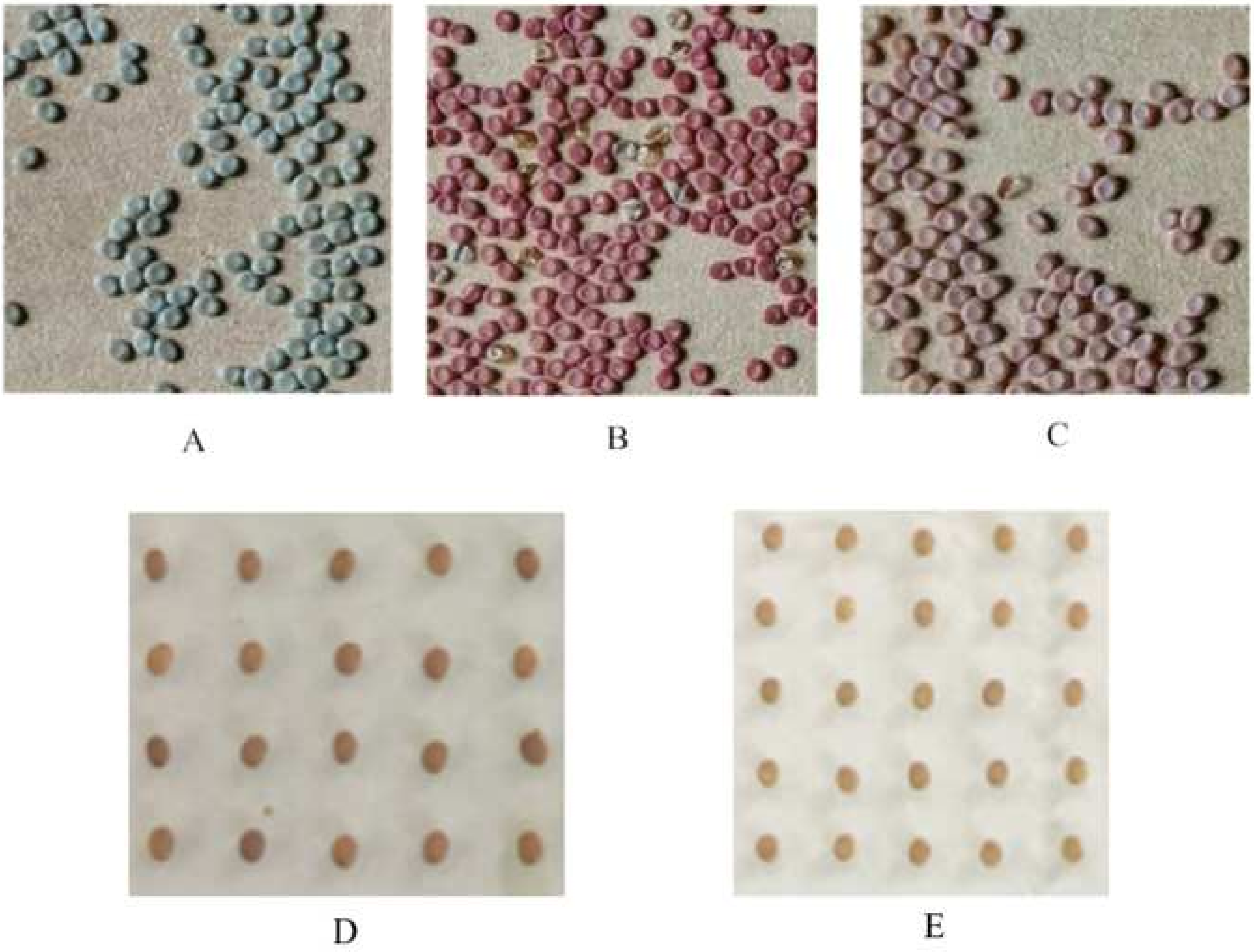

Figure

Click here to access/download;Figure;Fig 2.tif

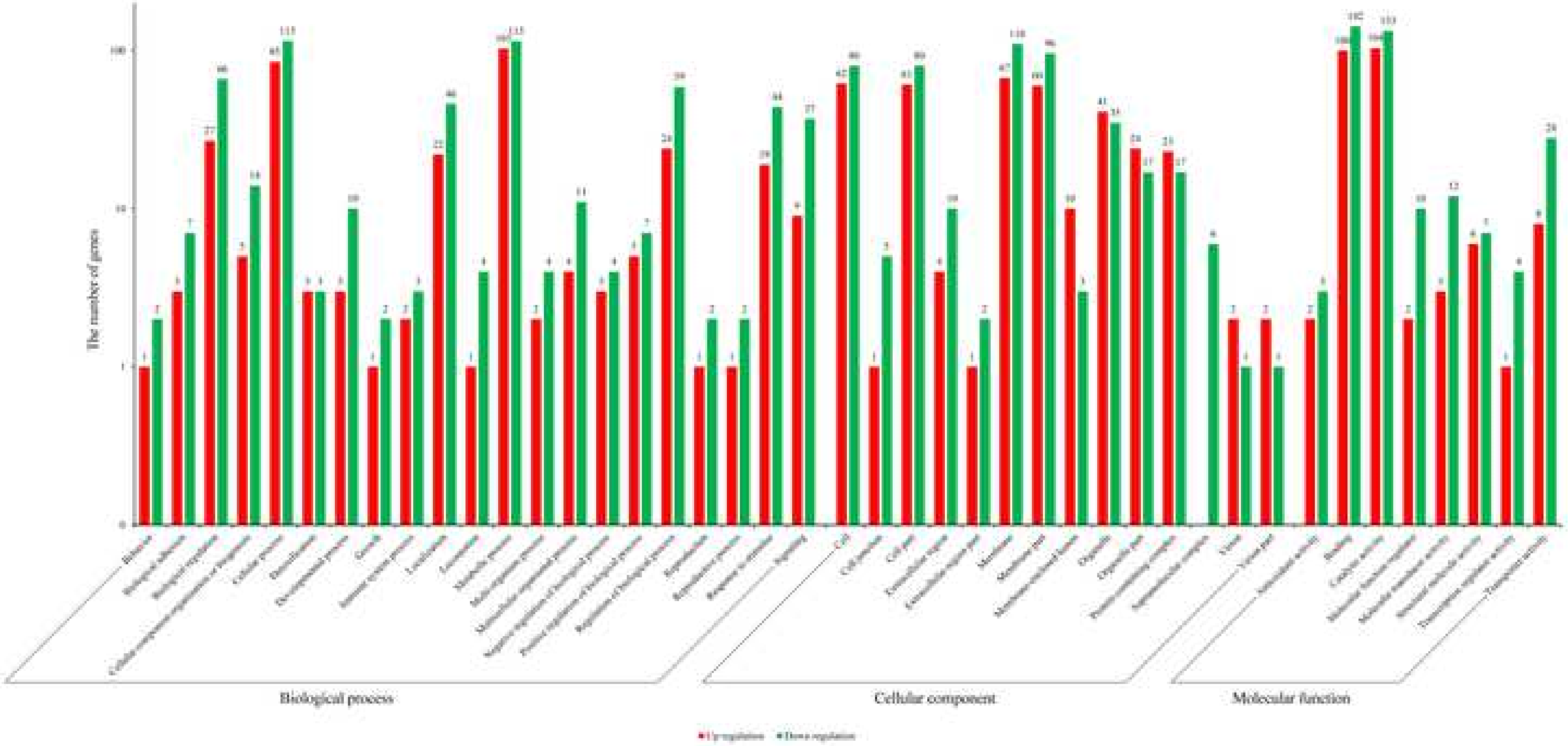

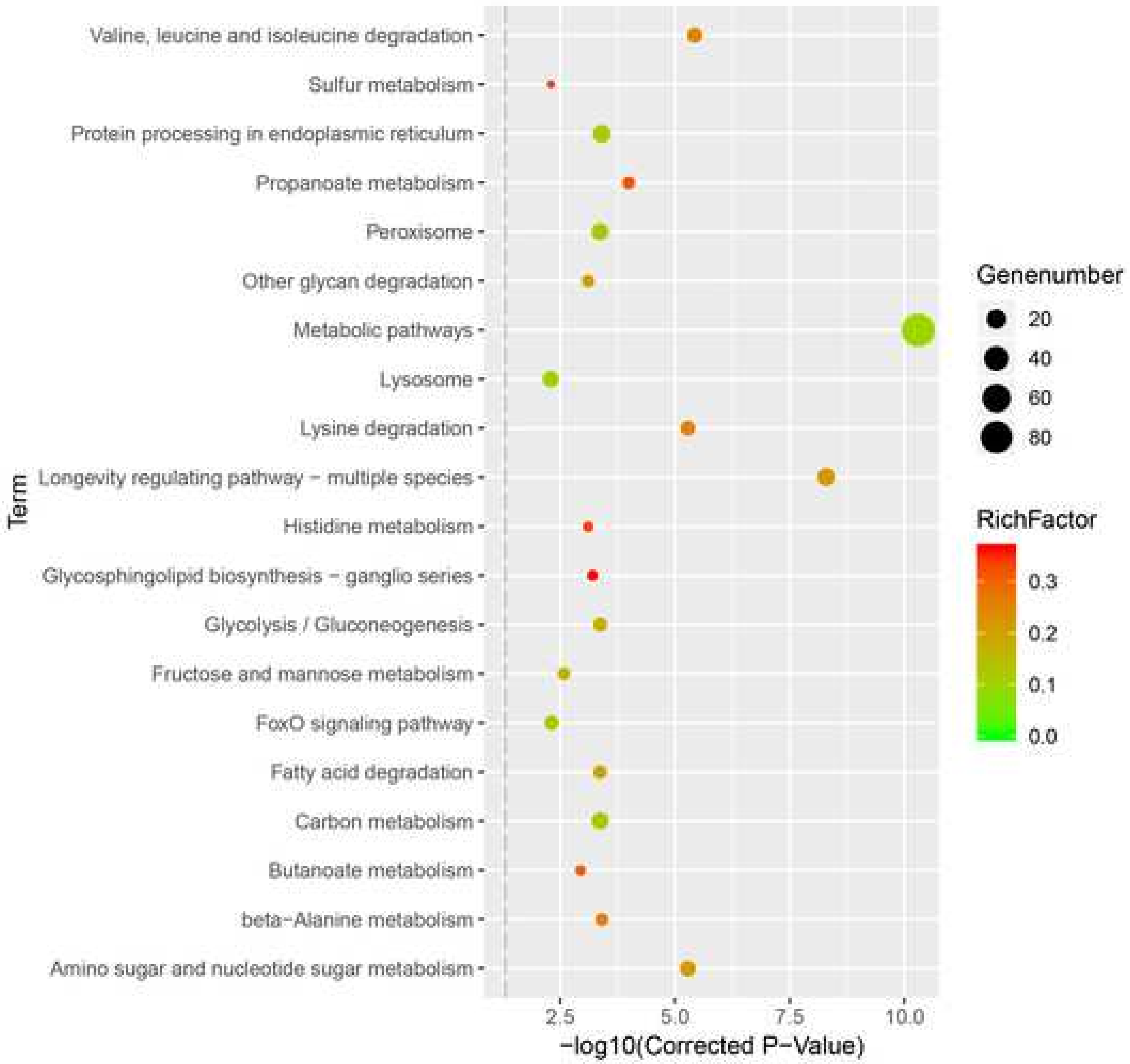
Valine, leucine and isoleucine degradation
Sulfur metabolism
Protein processing in endoplasmic reticulum
Propanoate metabolism
Peroxisome
Other glycan degradation
Metabolic pathways
Lysosome
Lysine degradation
Longevity regulating pathway − multiple species
Histidine metabolism
Glycosphingolipid biosynthesis − ganglio series
Glycolysis / Gluconeogenesis
Fructose and mannose metabolism
FoxO signaling pathway
Fatty acid degradation
Carbon metabolism
Butanoate metabolism
beta−Alanine metabolism
Amino sugar and nucleotide sugar metabolism
Term
2.5
5.0
7.5
10.0
−log10(Corrected P−Value)
Genenumber
20
40
60
80
RichFactor
0.3
0.2
0.1
0.0

Figure

Click here to access/download;Figure;Fig 4.tif

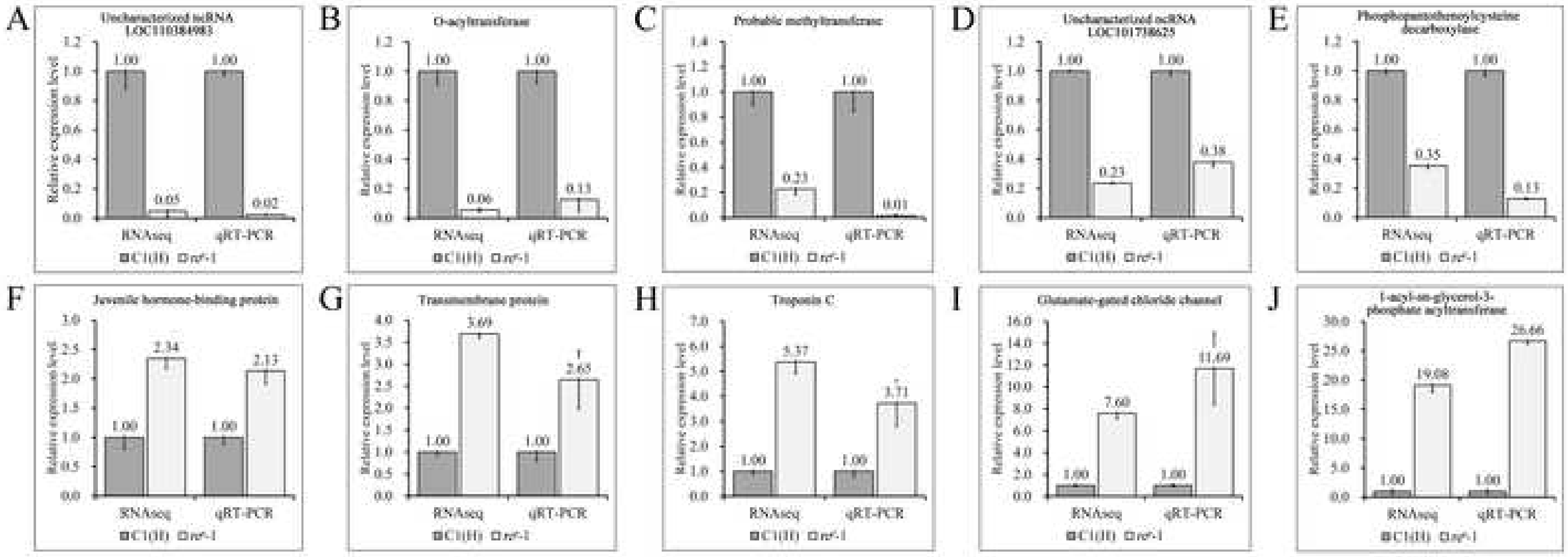

Figure
Click here to access/download;Figure;Fig 5.tif

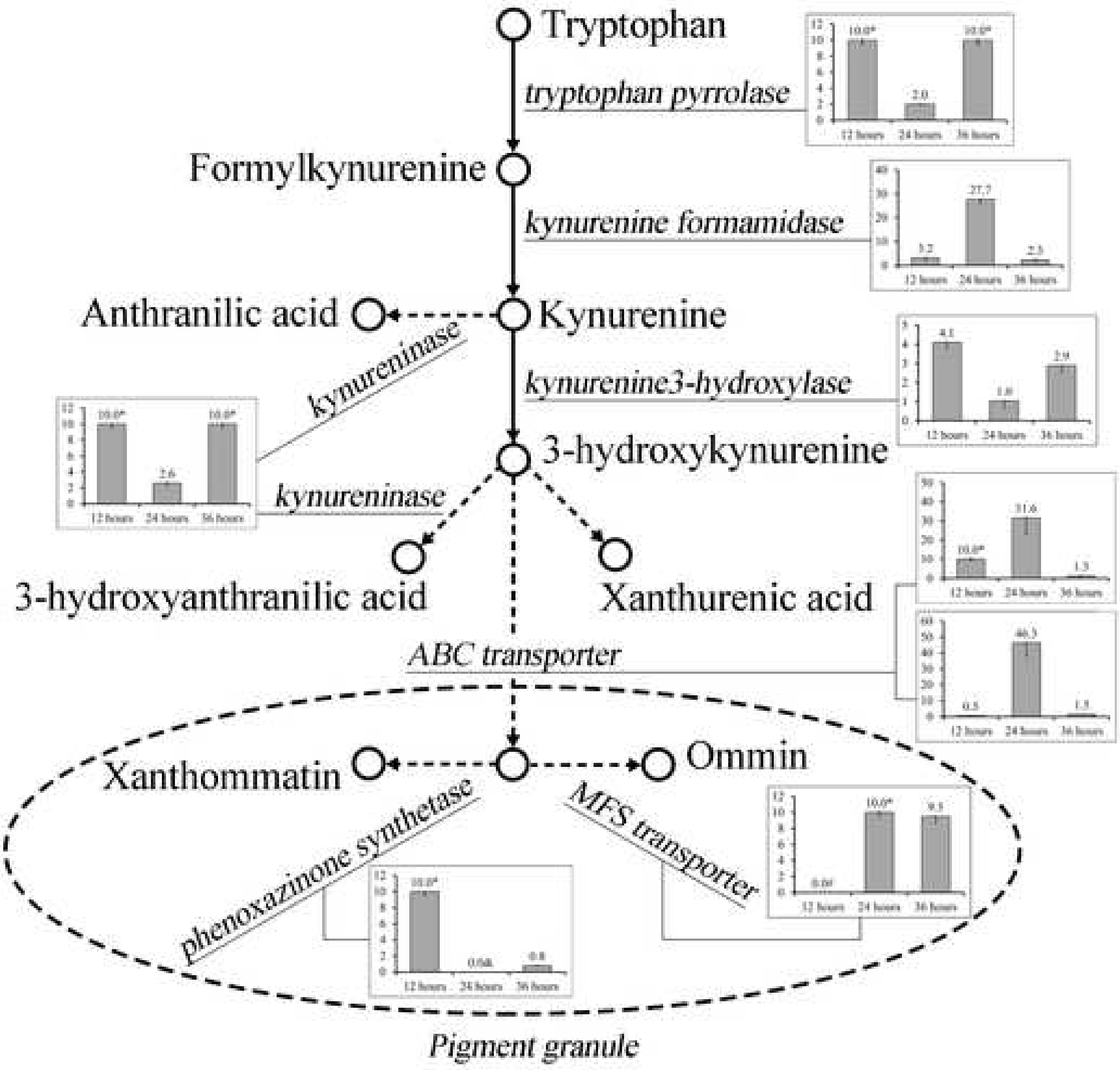

Supplement: Attachment — Submitted filename: PONE-D-20-03586_R2-jh.pdf [file pone.0237242.s012.pdf]
