# Supplementary material for: Transcriptome analysis of the eggs of the silkworm pale red egg (rep-1) mutant at 36 hours after oviposition
Source: PLoS One. 2020 Aug 7;15(8):e0237242. doi: 10.1371/journal.pone.0237242 (PMC7413551; doi:10.1371/journal.pone.0237242)
Supplement: Attachment — Submitted filename: Response to reviewers.doc [file pone.0237242.s010.doc]

Dear Editor,

Thanks to you and the reviewers for the valuable suggestions. We have carefully revised our [manuscript](https://fanyi.so.com/?src=onebox#manuscript) according to your suggestions. In addition, the language was improved by English editors at American Journal Experts. The response are listed below. We greatly appreciate your time and efforts to improve our manuscript for publication.

Sincerely yours

Qiaoling Zhao and Meina Wu

**To Editor:**

1. As currently written, the Methods section lacks sufficient details (and references) to allow for replication. I recommend looking at the relevant sections of the following papers for examples on the details needed for sequencing/quality control/quantification of the Illumina data.

**Response:** In the method section, we have supplemented the processing of RNA library construction and sequence assembly and references as well.

2. qPCR methods and data should include amplification efficiencies.

**Response:** We have modified the qPCR process in Method and supplemented amplification efficiencies.

3. Related to Reviewer 1 comments re the nomenclature used. If *re^p^*-1 is a derivative of the re mutant, why was the re mutant not included in the analyses?

**Response:** The *re^P^*-1 is derived from silkworm strain C1(H) with pale brown egg color, not from the *re* mutant. In this study we mainly focused on the mutant *re^p^*-1 and its wild type, C1(H) . The hybridization map between *re* and *re*^p^-1 mutants are shown below, which demonstrates that the *re*^p^-1 gene may be very closely related to the *MFS* gene (in reference 15). We will submit this article to you.

**Genetic relationship between *re^p^-*1 and *re* mutants**

When hybridized the *re^p^-*1 (as female) mutant with the *re* mutant (Fig. A), all the F1 generation eggs were pale red eggs. When F1 generation was self-bred to produce F2, all the eggs in F2 generation were red eggs. F2 generation was self-bred to produce F3 which appeared character segregation: red eggs, pale red eggs and transitional colored eggs . In contrast, when hybridized the *re* (as female) mutant with *re^p^-*1 the mutation(Fig. B), all the F1 generation eggs were red eggs. When F1 generation was self-bred to produce F2, all the eggs in F2 generation were red eggs. F2 generation were self-bred to produce F3 which also appeared character segregation, due to eggs are in the different degree of red color, and difficult to distinguish from red eggs and pale red eggs.

The eggs in F2 generation were all red which could be inferred that the gene controlling the *re^p^-*1 mutation was a recessive gene. The gene who controls the *re* mutationis *MFS* [14]. The above results showed that the gene controls the *re^p^-*1 mutant may be very closely related to the *MFS* gene.

4. It is not clear if qualitative differences were examined. Relatedly, since this is referred to as *re^p^*-1, is the same/similar mutation in the MFS transporter reported in the original Osanai-Futahashi manuscript present?

**Response:** In previous study[reference 15]，we found that in the *re^p^*-1 mutant the sixth exon of *MFS* gene lacks a 59bp fragment which is instead by a 14 bp sequence. The difference between *re^p^*-1 and *re* (Osanai Futahashi, 2012) is shown in the figure below.

E. The transmembrane domain analysis of *MFS* gene in wild type F. The transmembrane domain analysis of *MFS* gene in *re^p^* mutant G. The transmembrane domain analysis of *MFS* gene in *re* mutant (type1) H. The transmembrane domain analysis of *MFS* gene in *re* mutant (type2).

(Referance 15)

Line 357 states that the mutant is “unable to complete ommochrome synthesis because of gene mutations during the early stage..”. What are these mutations?

**Response:** These are white egg mutations which have been introduced in the introduction, and the specific mutation process of *w-*1, *w-*2 and *w-*3 in references 11, 12 and 13 respectively.

As written, it suggests that the mutations are qualitative. If not, this should be re-phrased so that is more clear that the phenotype is driven by quantitative differences in transcript expression, which could indicate potential changes to transcription factors. If so, were these examined?

**Response:** The *re^p^-*1 is a recessive gene in contrast to wt (see the first response to Reviewer #1). In previous study[reference 15]，we have analyzed the transcriptional expression level of *MFS* in *re^P^*-1, re and wt type at different period post oviposition. The results indicated that MFS expressed in high level both in *re^p^*-1 and wt without significant difference, while in re mutant MFS level was significantly low than that in *re^p^*-1 and wt (see the figure below).

(Referance 15)

5. Indicate in Figure 7 the genes that differ from WT, perhaps by addition of colors or some other type of marker. It is not clear in the current version where the differences occur.

**Response:** Now it’s changed to Figure 4. We have modified Figure 4 and carefully annotated it. We have explained in the Results (Differentially expressed genes related to ommochrome synthesis), in which the quantitative analysis of gene expression in the ommochrome synthesis and metabolism pathways were performed at 12 h, 24 h and 36 h after oviposition, respectively.

**To Reviewer #1:**

1 One is that the authors are using the name *re^p^*-1 for the newly found egg color mutant without offering basis that it is an allele of the re mutant. They refer to a paper that this mutant is controlled by a recessive gene on the autosome, but the paper could not be easily accessed at least by google scholar search. Nor the information on the chromosome number the gene is located is provided.

**Response:** The genetic model of *re^P^*-1 egg color trait and the genetic relationship between *re* and *re^p^*-1 mutants were as follows. And we will submit the article to you, which is in Chinese with an English abstract.

**The genetic model of egg color character of *re^p^-*1 mutant**

Hybridized the *re^p^-*1 mutant with normal variety p50 (orthogonal or backcross). all F1 generation laid brown eggs. And F2 generation self-hybridized with F1 generation which appeared character separation: brown eggs and pale red eggs which separation ratio was closed to 3:1( see table below from reference 15). Hybridized p50 with F1 generation (orthogonal or backcross), all the eggs were normal. Hybridized *re^p^-*1 mutant with F1 generation (orthogonal or backcross) which appeared character separation: brown eggs and pale red eggs which separation ratio was closed to 1:1. The genetic model indicated that the mutation of pale red eggs was controlled by an autosomal recessive gene.

**Genetic relationship between *re^p^-*1 and *re* mutant**

Hybridized the *re^p^-*1 (as female) mutant with the *re* mutation, all the F1 generation eggs were pale red eggs. When F1 generation was self-bred to produce F2, all the eggs in F2 generation were red eggs. F2 generation was self-bred to produce F3 which appeared character segregation: red eggs. Pale red eggs and transitional colored eggs; Hybridized the *re* (as female) mutant with *re^p^-*1 the mutation, all the F1 generation eggs were red eggs. When F1 generation was self-bred to produce F2, all the eggs in F2 generation were red eggs. F2 generation were self-bred to produce F3 which also appeared character segregation, due to eggs are in the different degree of red color, and difficult to distinguish from red eggs and pale red eggs.

The eggs in F2 generation were all red which could be inferred that the gene controlled the *re^p^-*1 mutation was recessive of the gene who controls the *re* mutation, MFS gene [14], The above results showed that the gene controls the *re^p^-*1 mutant may be very closely related to the *MFS* gene.

2. this paper needs more experiments and data before publishing in PLOS One.I suggest the authors at add the following data. Egg photos of C1 (H) and *re^p^*-1 strains at 36h and at least 1 time point each before and after 36h post oviposition. photos of adult eyes of C1 (H) and *re^p^*-1 strains complementation test with re mutant. If it is not an allele of re, linkage analysis for *re^p^*-1 (RAD-seq using different WT strain may be effective) conduct qRT-PCR for fig 6 or RNAseq for other stages (at the least, one point before 36h)

**Response:** We have added the photos of eggs of C1 (H), *re^P^*-1 and *re* (S1_Fig 1A,1B and 1C). Normal type C1( H) after 36 hours of oviposition . Pale red egg (*re^p^*-1) mutant after 36 hours of oviposition (S1_Fig 1D,1E) .Comparison of silkworm egg color, the genetic model of *re^p^*-1 egg color trait and the genetic relationship between *re* and *re^p^*-1 mutants have been added to the answer 1

3. The first sentence of the abstract should be deleted, because it has little relevance with the main subject of this paper. Figures 1, 2, 3 should be moved to Supplementary data.

**Response:** We have deleted the first sentence of the abstract and figures 1, 2, 3 have been moved to Supplementary data.

4.line 66-68"Eventually, two molecules of 3-hydroxy-kynurenine are synthesized by phenoxazinone synthetase (PHS) to produce lutein."This is incorrect. The authors seem to mistaken xanthommatin for lutein. Lutein is carotenoid, not ommochrome.

**Response:** It's translation error. We have changed lutein to xanthommatin.

5. "pyrrolasc" may be typo (unfamiliar term to me) figure 6 (seems to be the most important figure at present): Presenting only NCBI gene ID is not common. The name of the gene or should be stated. If it is not a previously studied gene, at least kind of the gene, such as non-coding RNA, and the chromosome number the gene is located should be stated.

**Response:**"pyrrolasc" has been changed to pyrrolase, and we have added gene name in comparison with gene ID in NCBI.

6. please state clearly in the text whether the expression data for 12 and 24 hours post oviposition were conducted by the authors for this manuscript (If so, the results should in the results section and stated clearly in the materials and methods section) or refers to data from a previous study. Also, the label for vertical axis should be clearly stated (is this expression data of *re^p^*-1 or WT?

**Response:** The expression data for 12 and 24 hours post oviposition, we obtained by experiments, not from references. We have added 12h and 24h contents in the materials and methods section, The label for vertical axis was a ratio (expression of *re^p^-*1 mutant / expression of wildtype). If the ratio> 2 or <0.5, it indicates that there are differences in expression quantity.

**To Reviewer #2**

1.In Materials and methods, line no 127 onwards, the numbering can be brought down, instead of running text as it confuses in continuous reading

**Response:** We have revised it according to your suggestion.

2. Flow chart can be made for the various processes involved in Quality check of the data and its analysis

**Response:** We have revised it to flow chart for clarity.

3. In references can be mentioned instead of et al like in 2nd, 8th, 11,12, 14, 15,20, 21, 32, 35 and 36.

**Response:** We've revised them and added the complete author name.
