# Supplementary material for: Transcriptome analysis of the eggs of the silkworm pale red egg (rep-1) mutant at 36 hours after oviposition
Source: PLoS One. 2020 Aug 7;15(8):e0237242. doi: 10.1371/journal.pone.0237242 (PMC7413551; doi:10.1371/journal.pone.0237242)
Supplement: Attachment — Submitted filename: Response to Reviewers.docx [file pone.0237242.s011.docx]

Dear Editor,

Thank you and the reviewers for the valuable suggestions. We have carefully revised our [manuscript](https://fanyi.so.com/?src=onebox#manuscript) according to your suggestions. In addition, the language was improved by English editors at American Journal Experts. The response are listed below. We greatly appreciate your time and efforts to improve our manuscript for publication.

Sincerely yours

Qiaoling Zhao and Meina Wu

**To Editor:**

1. From the authors information on the result of complementation test, I assume there is a possibility of *re^p^*-1 is an individual allele of re mutant with independent origin (though the coloration of F2 and F3 eggs may not be explained only by Bmre gene). However, this information is not provided in the revised text but only in the answers to reviewer and editor. These are very important information and the original paper is not easily accessible to non-chinese researchers. The genetic relationship between *re^p^*-1 and re (i.e. the results of complementation test), and also the mutation in Bmre gene in *re^p^*-1 mutant should be described and explained in the last paragraph of introduction section so that the general readers are given enough background information to evaluate the results of this study.

**Response:** We have added Genetic relationship between *re^p^*-1 and re mutants and The genetic model of egg color character of *re^p^*-1 mutant to the introduction section.

2. Bm-re is not a transcription factor or a signal transducer, but a transporter. Since the authors stand on the basis that *re^p^*-1 is an allele of re and there is enough possility, they should discuss the link between the mutation in Bm-re gene in *re^p^*-1 mutant and the differential expression of genes between *re^p^*-1 and C1H, if they aim to say the differential expression of these genes are caused by Bm-re mutation. However, there may be a possibility that differential gene expression detected in this study (i.e. Fig. 3) is caused by a locus other than Bm-re. Expression analysis between individuals obtained by several rounds of backcross may answer this.

**Response:** Thank you for your suggestions. Our recent experiments based on positional cloning indicated that mutation of another gene rather than Bm-re might be the key of *re^p^*-1 mutant. We will publish these results later.

3. In the last sentence of introduction, the authors state that “The study aims to build a foundation for understanding the formation mechanism of *re^p^*-1 mutants and provides a theoretical basis for the study of the molecular mechanism of egg color formation.”If we stand on the basis that *re^p^*-1 is an allele of re, the most simple explanation for color change in *re^p^*-1 is the lack of a pigment precursor due to the disruption of Bmre gene.In the current version of the manuscript, the results of this study do not seem to add information on the cause of color change in the *re^p^*-1 mutant nor egg color formation.

Response: In this manuscript, we mainly analyzed differentially expressed genes affected by the main gene. About the key gene of *re^p^*-1 mutant, relative researches are ongoing. And our recent results did not support Bm-re as the main gene of *re^p^*-1 mutant. Relative data are under collating for publishing. In the following researches, conclusions in this manuscript will play important role in exploring the formation mechanism of the mutant character of *re^p^*-1 mutant especially the molecular mechanism of egg color formation. Then we think this study pays a foundation for understanding the formation mechanism of *re^p^*-1 mutants and provides a theoretical basis for the study of the molecular mechanism of egg color formation.

4. In the revised Figure 3, it is preferred that the name of the gene in addition to accession number is provided in the figure panel or in the figure legend.

**Response:** we have changed the accession number to the gene name in Figure 4.

5. Photos of eggs are better included in the main figure than supplementary figure.

**Response:** Photos of eggs has been removed to the main figure.
