# Supplementary material for: Transcriptome analysis of the eggs of the silkworm pale red egg (rep-1) mutant at 36 hours after oviposition
Source: PLoS One. 2020 Aug 7;15(8):e0237242. doi: 10.1371/journal.pone.0237242 (PMC7413551; doi:10.1371/journal.pone.0237242)
Supplement: Attachment — Submitted filename: Response to Reviwers.doc [file pone.0237242.s013.doc]

**Dear Editor and Reviewers:**

Thank you for your valuable suggestions. We have modified the copyediting issues and unclear word usage as you marked. The responses are listed below. We greatly appreciate your time and efforts to improve our manuscript for publication.

Sincerely

Qiaoling Zhao

To Editor and Reviewers:

1.what is meant by orthogonal or backcross? please clarify？

Response: Hybridizing the *re^p^*-1mutant with normal variety p50 (orthogonal or 96 backcross), orthogonal means the *re^p^*-1mutant as female crossed with the normal variety p50, and the backcross means the *re^p^*-1mutant as male crossed with the normal variety p50, the offspring is F1 generation.

2. what type of reads were generated? 150-bp paired end reads?

Response: Yes. 150-bp paired end reads data of the processing group and the control group library was obtained.

3. what software was used to do this? A corresponding reference should be added as well.

Response: Trinity-v2.8.4. and we have added the reference [16].

4. which version of Trinity was used? what parameters were used? Add in the reference for Trinity.

Response: The clean reads were assembled by using the short reads assembler Trinity-v2.8.4.

All experiments were performed with Trinity using parameters: minimum contig length of 100 bases and average fragment length of 300 bases. and we have been added the reference“Grabherr MG, Haas BJ, Yassour M, Levin JZ, Thompson DA, et al. Full-length transcriptome assembly from RNA-Seq data: without a reference genome. Nat Biotechnol. 2011; 29: 644-U130.”

5. what e value cut-off was used for BLASTx?

Response: E-value cut-off =10^-5^

6. which est scan software? proivde a reference?

Response: ESTScan software 2.1 and we have added the reference“Iseli C, Jongeneel CV, Bucher P. ESTScan: a program for detecting, evaluating, and reconstructing potential coding regions in EST sequences. Proc Int Conf Intell Syst Mol Biol. 1999; 138–148.”

7. analyzed with which software?

Response: ESTScan software 2.1.

8. what software/program was used to examine RPKM?

Response: The RSEM program of Trinity software-v2.8.4 was used to standardize the expression level of each gene.

9. should this be 10 μM?

Response: Thanks for your suggestion and we have modified it.

10. this method does not analyze melt curves

Response: We have been modified to “the 2-ΔΔCt method was used to examine differential expression of the genes of interest”.

11. what is meant by Mega raw reads?

Response: We have modified to“Raw reads”.

12.is there an accession number for this?

Response: The accession number is ASM15162v1.

13. define MFS?

Response: MFS (major facilitator superfamily).

14. white and scarlet?

Response: Yes. “two ABC transporters responsible for transporting 3-hydroxy kynurenine to pigment granules.” The pigment granules which includes white and scarlet.

16. clarify what is meant by "sperm". Does the egg shell contribute to sperm protection and/or sperm exchange? As written, the sentence suggests both.

Response: Thanks for your suggestion. We have deleted it.

17. Perhaps indicate which gene is different.

Response: We have added gene 101746088 in the manuscript. It was a vitellogenin gene which was down-regulated in the light red egg mutants.

18. define CPGs?

Response: CPGs (cuticular protein genes) which can affect the formation of insect epidermis.

19. fix spacing issues - (A) Normal type C1(H). (B) Red egg (re) mutant...et

Response: Thank you for your suggestion. We have fixed spacing issues.

20. what is meant by intensiveness?

Response: The intensiveness means a lot of enrichment rich factor match with the pathway.

21. what is "number in title". Clarify.

Response: We have been modified the description to “The LOC number represented the gene ID in NCBI. All genes were closely consistent between transcriptome analysis and qRT-PCR verification, which marked with red box.

22. megabases? gigabases?

Response: Thank you for your valuable suggestion. We have modified to megabases and gigabases.
